# Rapid Determination of 30 Polyphenols in Tongmai Formula, a Combination of Puerariae Lobatae Radix, Salviae Miltiorrhizae Radix et Rhizoma, and Chuanxiong Rhizoma, via Liquid Chromatography-Tandem Mass Spectrometry

**DOI:** 10.3390/molecules22040545

**Published:** 2017-03-29

**Authors:** Fu-Rong Wang, Ying Zhang, Xin-Bao Yang, Chun-Xu Liu, Xiu-Wei Yang, Wei Xu, Jian-Xun Liu

**Affiliations:** 1 State Key Laboratory of Natural and Biomimetic Drugs, Department of Natural Medicines, School of Pharmaceutical Sciences, Peking University Health Science Center, Peking University, No. 38, Xueyuan Road, Haidian District, Beijing 100191, China; frwang1983@163.com (F.-R.W.); high-xu@163.com (W.X.); 2 Institute of Bascic Medical Sciences, Xiyuan Hospital, China Academy of Chinese Medical Sciences, Beijing 100091, China; zhyingde@sina.com (Y.Z.); xbyang0718@163.com (X.-B.Y.); liuchunxu00000@126.com (C.-X.L.); 3 School of Basic Medical Science, Beijing University of Chinese Medicines, Beijing 100029, China; 4 Faculty of Traditinal Chinese Medicine, Shenyang Pharmaceutical University, Shenyang 110016, Liaoning, China

**Keywords:** Tongmai formula, LC-ESI-MS/MS, polyphenol, Puerariae Lobatae Radix, Salviae Miltiorrhizae Radix et Rhizoma, Chuanxiong Rhizoma

## Abstract

Tongmai formula (TMF) is a herbal preparation composed of three traditional Chinese medicinal materials: Puerariae Lobatae Radix (Gegen), Salviae Miltiorrhizae Radix et Rhizoma (Danshen) and Chuanxiong Rhizoma (Chuanxiong). It has been used to treat cardiovascular diseases for decades. To develop a reliable and convenient analytical method for a comprehensive determination of polyphenols in TMF and the ascertainment of their chemical correlations with its herbal components, a method combining high-performance liquid chromatography with electrospray ionization tandem mass spectrometry (LC-ESI-MS/MS) was developed and validated for rapid determination of 30 polyphenols in TMF and its three herbal components. The chromatographic separation was carried out on a Chromolith Fastgradient RP-18 endcapped 50-2 column using an optimized gradient elution. Statistical analysis of obtained data demonstrated that the method had a desirable linearity, precision, and accuracy, as well as excellent sensitivity. The obtained results indicated that, among the 30 polyphenols in TMF, 22 originated from Gegen, 6 originated from Danshen, and 2 originated from Chuanxiong. The major polyphenols in TMF have been identified as puerarin, mirificin, salvianolic acid B, salvianic acid A, 3’-hydroxypuerarin, 3’-methoxypuerarin, and salvianolic acid A, with a combined contribution of 19.2% of the preparation. The development and validation of this method will greatly facilitate future pharmacological studies of TMF and its herbal components, as well as polyphenols in cardiovascular therapies.

## 1. Introduction

Polyphenols are secondary metabolites widely existing in plants but cannot be synthesized by animals and human beings [1]. They are a family of phenolic compounds with one or more aromatic rings bearing one or more acidic hydroxyl groups. The polyphenol family can be classified by the backbone structures as phenolic acids (derivatives of benzoic acid and cinnamic acid), stilbenes, lignans, and flavonoids. The sub-family of flavonoids can be further classified by the structures of their heterocyclic C-rings into flavanols, flavanones, flavonols, anthocyanidins, flavones, isoflavonoids, and homoflavones [2]. It has been shown that polyphenols can improve endothelial function [3], and inhibit platelet aggregation [4], antithrombotic [1], and anti-inflammatory [5] properties. On the basis of these findings, polyphenols may play important roles in the prevention and treatment of cardiovascular diseases.

Tongmai formula (TMF) is a traditional Chinese medicine listed in the Ministerial Drug Standard of China for promoting blood circulation and removing blood stasis. It consists of three traditional Chinese medicinal materials including Puerariae Lobatae Radix (Gegen, roots of *Pueraria lobata* (Willd.) Ohwi), Salviae Miltiorrhizae Radix et Rhizoma (Danshen, roots and rhizomes of *Salvia miltiorrhiza* Bge.), and Chuanxiong Rhizoma (Chuanxiong, rhizomes of *Ligusticum chuanxiong* Hort.). It has been proved that polyphenols are the main active components in these herbal components responsible for TMF’s beneficial functions for cardiovascular diseases.

Isoflavones and their glycosides are major bioactive constituents in Gegen. Their pharmacological actions on cardiovascular system have been extensively investigated [6]. The major isoflavone, puerarin, is supposed to account for the pharmacological actions of Gegen according to its effectiveness on relevant cell lines in vitro and animal models in vivo [7,8]. Other isoflavones, such as daidzin, daidzein, and formononetin, also exert various activities on cardiovascular diseases [7,9,10]. Danshen has been used primarily in traditional Chinese medicine for the remedy of coronary heart diseases, particularly for angina pectoris and myocardial infarction. Phenolic acids including caffeic acid monomers and oligomers are major constituents in water-soluble extract of Danshen. The most prominent cardiovascular effects of these phenolic acids are antioxidation [11], anti-blood coagulation [12], and vasodilation [13]. Ferulic acid, the most abundant phenols in Chuanxiong, has been declared to be responsible for the efficacy of Chuanxiong on cardiovascular diseases. It shows antioxidative activity in vitro, inhibits platelet aggregation, prevents thrombus formation [14], exerts anti-hypertension effects [15,16], and reduces both triglyceride and total cholesterol plasma levels in vivo [14,16].

Although high-performance liquid chromatography (HPLC) fingerprint spectra have shown that polyphenols are the main constituents in TMF [17] and over 20 kinds of isoflavones have been isolated and identified from TMF [18,19], their exact contents are not clear yet. Due to their essential pharmacological activities, reliable and convenient analytical methods are highly desired for the studies of the polyphenols. However, the complex molecular composition and the molecular structural resemblance of these compounds have greatly challenged such efforts in efficient separations and sensitive detections. Up to now, very few analytical methods have been reported to practically achieve these goals.

An ultraviolet (UV) detector is commonly utilized for the detection of polyphenols in virtue of their high-quality UV absorption. There have been many reports in which thin layer chromatography, HPLC, or capillary electrophoresis coupled with UV was used to determine constituents in the herbal components of TMF [20,21,22,23]. However, due to the insufficient selectivity and sensitivity of UV, these technologies suffered from a long analysis time [20,21], low resolution, low sensitivity [20], and/or few analytes [20,22,23] in case of simultaneous determinations.

**Figure 1:**
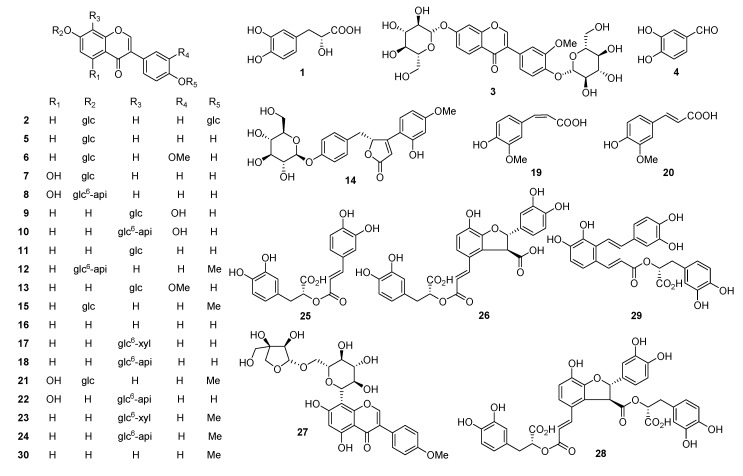
The chemical structures of 30 polyphenols isolated from Tongmai formula (TMF).

In this study, we report the development of a method combining high-performance liquid chromatography with electrospray ionization tandem mass spectrometry (LC-ESI-MS/MS) to analyze the polyphenols in TMF and its herbal components. The separation of the complex polyphenol system was achieved by HPLC with a silica-based monolithic column and an optimized elution condition; the detection of the analytes was achieved with a triple quadrupole tandem mass spectrometer (MS/MS) with electrospray ionization (ESI) and multiple reaction monitoring (MRM). This method has provided comprehensive qualitative analysis on TMF: a total of 30 polyphenols as shown in Figure 1 were identified, and their herbal originals were also ascertained. In addition, reliable quantitative analysis on TMF, e.g., the exact contents of 30 polyphenols, was achieved with this method. The development and validation of this method will greatly facilitate future pharmacological studies of TMF, its herbal components, and polyphenols in cardiovascular therapies.

## 2. Results and Discussion

### 2.1. Method Development

Among all 30 target phenols, phenolic acids could only obtain satisfactory product ions in a negative ionization mode. For other components, almost all were flavones, as they could be cracked easily in both positive and negative ionization mode, the superior ionization mode for analysis was selected by comparing the sensitivity and stability of their precursor/product ion transitions in each mode. The comparison results claimed an apparent relationship between the structure of the flavonoid glycosides and their superior ionization mode. A positive mode was shown to be more suitable for most flavonoid glycosides except for those with the position of R_3_ substituted (**9**–**11**, **13**, **17**, **18**, **22**–**24**), which displayed better sensitivity in a negative ionization mode. Quantitative transitions from precursor to product ions (MRM transitions) were identified for each compound as those described in Table 1, in which 11 phenols (**2**, **3**, **5**–**8**, **12**, **14**–**16**, **21**) were optimized in +ESI mode and the other 19 phenols (**1**, **4**, **9**–**11**, **13**, **17**–**20**, **22**–**30**) were in −ESI mode. Typical chromatograms of individual MRM transitions for 30 compounds in the extract of TMF or Puerariae Lobatae Radix, Salviae Miltiorrhizae Radix et Rhizoma, and Chuanxiong Rhizoma are shown in Figure S1.

Since there are multiple pairs of isomers in target components list (such as **19**/**20**, **6**/**21**, **17**/**18**, **23**/**24**, **10**/**22**), fine separation by HPLC was essential for accurate quantitation but would usually prolong the duration of analysis. A silica-based monolithic column was eventually employed to achieve a fast and efficient separation. Due to its rigid and porous structure, it enables higher solvent flows, shorter analysis times, and faster column equilibration between runs without increased back pressure or loss in efficiency, as compared with conventional packed columns [24]. Under the condition of optimized gradient mobile phase, isomers were separated on a Chromolith Fastgradient RP-18 endcapped 50-2 column (Merck Chemicals, Darmstadt, Germany) satisfactorily. A mobile phase containing 0.1% formic acid utilized in negative ESI mode greatly improved the peak shapes of phenolic acids, especially salvianolic acid B and rosmarinic acid, from serious tailing, though commonly bringing disadvantages to the sensitivity of analytes by reducing their ionization efficiency.

### 2.2. Method Validation

#### 2.2.1. Linearity, Lower Limit of Detection, and Lower Limit of Quantification

The results of the investigation on linearity, regression equation, correlation coefficient (*r*), the lower limit of detection (LLOD), and the lower limit of quantification (LLOQ) are listed in Table S1. The correlation coefficient values (*r* ≥ 0.9990) indicated good correlations between the sampling amount on-column and their peak area within the test ranges. The LLOD was calculated corresponding to *S*/*N* 3:1 and the LLOQ was calculated corresponding to *S*/*N* 10:1. Fast separation and well optimized mass parameters brought superior sensitivity to the overall LLODs less than 37.06 pg.

#### 2.2.2. Precision, Repeatability, and Stability

Intra- and inter-day variations were chosen to determine the precision of the analysis method. For intra-day variability test, the mixed standard solutions were analyzed for six replicates within 1 day. For inter-day variability test, the solutions were examined in triplicates for three consecutive days. Variations were expressed via relative standard deviation (RSD). The precision of MS/MS peak area measurements was found to be less than 3.16% for all target polyphenols (Table S1). To confirm the repeatability, six independently prepared samples of TMF extract were analyzed by the proposed method. Based on six replicate injections, the repeatability (RSD) of the proposed method were in the range of 1.56%–4.55%. The stability was measured as 0.45%–5.06% (*n* = 6) by analyzing TMF samples in an autosampler at an interval of 12 h (0, 12, and 24 h).

#### 2.2.3. Recovery

Recoveries for all analytes were determined using a standard addition method in which three levels analyses of the spiked samples were extracted and then run according to the proposed pretreatment and measure procedure within the same day. The results are summarized in Table S2. The recoveries were within the range of 93.90%–105.45% and the overall RSD values from three replicate injections were less than 4.88%, demonstrating the method’s good recovery and precision.

### 2.3. Content Determination

**Figure 2:**
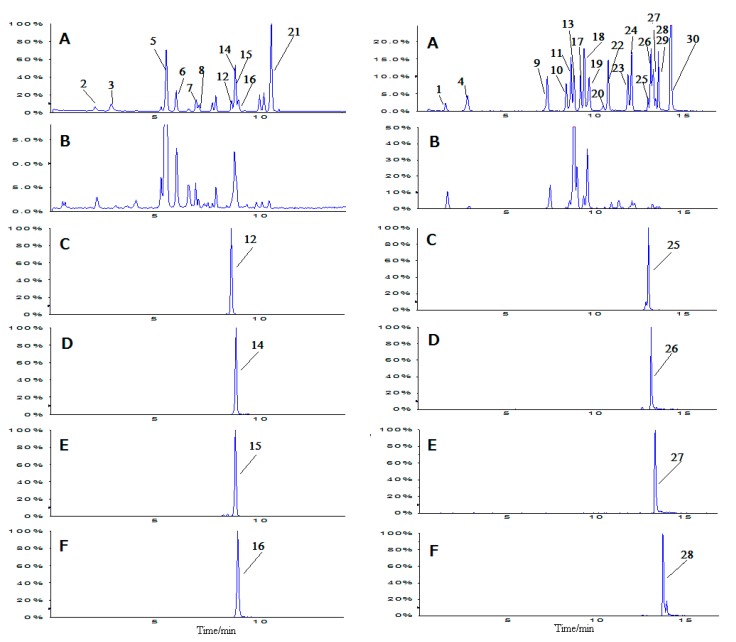
HPLC-MS/MS total ion current chromatograms (TIC) of mixed references (**A**) and Tongmai formula extracts (**B**) obtained in positive (left side) or negative (right side) ion ESI mode. MRM profiles of components overlapped in TIC spectrum were illustrated separately (**C**, **D**, **E**, and **F**).

HPLC-MS/MS total ion current (TIC) chromatograms of mixed references and TMF preparation obtained in negative or positive ion ESI mode are shown in Figure 2. Several extracted ion chromatograms (XIC) are also separately illustrated to distinguish the overlapped peaks in TIC. The most abundant polyphenols in TMF, each of which occupied over 10 mg/g in TMF preparation, were identified as puerarin, mirificin, salvianolic acid B, salvianic acid A, 3’-hydroxypuerarin, 3’-methoxypuerarin, and salvianolic acid A. These compounds accounted for 19.2% of the total content of TMF and 86.1% of all quantified phenols.

### 2.4. Chemical Correlation between TMF and Its Ingredient Herbs

The three medicinal materials were analyzed by the developed HPLC-ESI-MS/MS method to identify the attributions of these phenols and evaluate the influence of co-decocting processing on their contents. Comparing the retention time and mass data of precursor/product ion transitions of 30 quantitative polyphenols in TMF, it was found that 22 compounds (**2**, **3**, **5**–**18**, **21**–**24**, **27**, and **30**) originated from Gegen, 6 compounds (**1**, **4**, **25**, **26**, **28**, and **29**) originated from Danshen, and 2 compounds (**19** and **20**) originated from Chuanxiong. The contents of all investigated common polyphenols in TMF preparation and in each of its herbal components are shown in Table 2, Table 3 and Table 4. Mixed-decotion of TMF significantly enhanced the contents of **16** and **30**, which were 1.6- and 1.7-fold higher than the corresponding components in the extract of Gegan, respectively (*p* < 0.001). Apart from that, the contents of 16 polyphenols in TMF slightly changed, including 6 compounds (**9**, **11**, **13**, **24**, **27**, and **19**) up and 10 compounds (**2**, **3**, **5**–**8**, **21**, **25**, **26**, and **28**) down from its herbal components. The founding demonstrated that the concomitant preparation of these three herbs brought little affect on contents of most phenols except **16** (daidzein) and **30** (formononetin), which are aglycones of most flavonoid glycosides in Gegen. An increased amount of puerarin (**11**) contributed largely to an enhanced total concentration of 22 common components in TMF to 170.59 mg/g from that in Gegan at 159.56 mg/g. Some physicochemical factors might have accelerated the hydrolyzation of flavonoid glycosides during the mixed boiling procedure. Moreover, improved dissolution could have been another effective factor achieved with the help of other constituents in Danshen and Gegen during co-decoction in view of a relatively lower water solubility of aglycons and *C*-glycosides.

## 3. Experimental Section

### 3.1. Chemicals and Materials

Reference standards, daidzein-7,4’-di-*O*-β-d-glucopyranoside (**2**), 3’-methoxy-daidzein-7,4’-di-*O*-β-d-glucopyranoside (**3**), protocatechuic aldehyde (**4**), daidzin (**5**), 3’-methoxydaidzin (**6**), genistin (**7**), ambocin (**8**), 3’-hydroxypuerarin (**9**), 3’-hydroxymirificin (**10**), puerarin (**11**), formononetin-7-*O*-β-d-apiofuranosyl-(1→6)-*O*-β-d-glucopyranoside (**12**), 3’-methoxypuerarin (**13**), (±)-puerol B-4’-*O*-β-d-glucopyranoside (**14**), ononin (**15**), daidzein (**16**), 6”-*O*-β-d-xylosylpuerarin (**17**), mirificin (**18**), *trans*-ferulic acid (**20**), sissotrin (**21**), genistein-8-*C*-apiofuranosyl-(1→6)-*O*-β-d-glucopyranoside (**22**), formononetin-8-*C*-β-d-xylopyranosyl-(1→6) -*O*-β-d-glucopyranoside (**23**), formononetin-8-*C*-β-d-apiofuranosyl-(1→6)-*O*-β-d-glucopyranoside (**24**), biochanin A-8-*C*-β-d-apiofuranosyl-(1→6)-*O*-β-d-glucopyranoside (**27**), and formononetin (**30**) were all isolated from Tongmai formula extract by the authors [18,19]. The structures of these compounds were fully characterized by nuclear magnetic resonance (NMR) spectroscopy and mass spectrometry (MS). All reference substances were assayed by HPLC/diode-array detection (DAD), and their purities were over 98% on the basis of HPLC-UV determination. Reference standards, salvianic acid A (**1**), rosmarinic acid (**25**), and lithospermic acid (**26**) were purchased from Tianjin Yifang Science & Technology Co. Ltd. (Tianjin, China). Reference standards, *cis*-ferulic acid (**19**) and salvianolic acid B (**28**), were purchased from the National Institutes for Food and Drug Control (Beijing, China). Salvianolic acid A (**29**) (purity > 95%) was kindly provided by the Beijing Bencaotianyuan Institute of Pharmatech (Beijing, China). The chemical structures of Compounds **1**–**30** are shown in Figure 1. Medicinal materials of Gegen (No. 200910PL), Danshen (No. 200910SM) and Chuanxiong (No. 200910LC) were kindly provided by Shenwei Medicine Ltd. Co. (Shijiazhuang, China). They were authenticated by Prof. Xiu-Wei Yang of School of Pharmaceutical Sciences of Peking University (Beijing, China). Their voucher specimens have been kept at the Herbarium of State Key Laboratory of Natural and Biomimetic Drugs (School of Pharmaceutical Sciences, Peking University, Beijing, China).

Methanol (MeOH), acetonitrile (MeCN), and formic acid (FA) used in HPLC-ESI-MS/MS analysis were of HPLC grade and were purchased from Fisher Scientific (Springfield, NJ, USA). Deionized H_2_O (18 MΩ/cm) was generated in-house using a Milli-Q System from Millipore Corporation (Billerica, MA, USA). All other solvents used were analytical-grade commercial preparations (Beijing Chemical Works, Beijing, China).

### 3.2. Preparation of Standard Solutions

Individual stock solutions (containing 1.0 mg/mL or 10 mg/mL) were prepared in MeOH using reference substances. Appropriate aliquots of individual stock solutions were mixed and diluted with 65% aqueous MeOH to make a mixed standard solution at concentrations ranging from 2 to 6400 µg/mL, and used for recovery tests. Working standard solutions were prepared by diluting the mixed standard solution with MeOH–MeCN–H_2_O (5:5:90, *v*/*v*/*v*).

### 3.3. Sample Preparation

Extraction processing was conducted following the protocol of TMF recorded in Ministerial Drug Standard of China [25]. One gram each of Gegen, Danshen, and Chuanxiong were mixed together and then extracted with boiling water twice (1.5 h and 1 h, respectively) to obtain the extracts. The combined extracts were filtrated and then lyophilized by a freeze-dryer (LGJ0.5 type, Four-ring Science Instrument Plant Beijing Co., Ltd., Beijing, China) to obtain the powdered extract of TMF.

About 1 g each of Gegen, Danshen, and Chuanxiong were accurately weighed, severally. Then, the preparation following the identical procedure of TMF, which afforded the extracts of Gegen, Danshen, and Chuanxiong, respectively.

To prepare the sample solutions, the above dried extracts were dissolved in 65% aqueous MeOH (30 mL) by ultrasonic treatment for 30 min, respectively. Then, the solution was centrifuged at 3000 rpm for 10 min. Finally, each 1 mL of the supernatant was diluted 1000 times by the solvent of MeOH–MeCN–H_2_O (5:5:90, *v*/*v*/*v*) to obtain the sample solutions for HPLC-MS/MS analysis.

### 3.4. HPLC-MS/MS Analysis

HPLC was performed using an Agilent 1200 series liquid chromatography system (Agilent Technologies, Palo Alto, CA, USA) equipped with a quaternary pump, an autosampler, and a column compartment. A Chromolith Fastgradient RP-18 endcapped 50-2 column (50 mm × 2 mm, 5 µm; Merck Chemicals, Darmstadt, Germany) was operated at a flow rate of 0.300 mL/min. The mobile phase consisted of MeOH–MeCN (1:1, *v*/*v*) containing 0.1% (*v*/*v*) FA (A) and water containing 0.1% (*v*/*v*) FA (B) was used for negative ion ESI mode (for analysis of Compounds **1**, **4**, **9**–**11**, **13**, **17**–**20**, and **22**–**30**) with gradient elution: 0–1 min, 8% A; 1–9 min, 8%–30% A; 9–11 min, 30%–80% A; 11–11.01 min, 80%–8% A; 11.01–19 min, 8% A. The same mobile phase as above was used for positive ion ESI mode (for analysis of Compounds **2**, **3**, **5**–**8**, **12**, **14**–**16**, and **21**) with gradient elution: 0–8 min, 10%–40% A; 8–12 min, 40%–75% A; 12–12.01 min, 75%–10% A; 12.01–20 min, 10% A. The column temperature was 10 °C for negative ion ESI mode and 40 °C for positive ion ESI mode. An aliquot of 5 µL of each sample solution was injected. Mass spectrometry was performed using an API 4000 Q-TRAP triple-quadrupole mass spectrometer (Applied Biosystems/MDS Sciex, Foster City, CA, USA) equipped with a Turbo ionspray interface. The instrument was operated in negative and positive ion ESI mode, with MS/MS transitions monitored during liquid chromatography separation in MRM mode. Selection and tuning of precursor/product transitions, as well as analyte-dependent parameters of DP (declustering potential) and CE (collision energy) were performed using direct infusion of individual standard solutions in MeOH at a concentration of 1 mg/mL. The optimized parameters of ion source were as follows: ion source gas 1 and gas 2, 50 psi; curtain gas, 12 psi; collision gas, high; source temperature, 500 °C; ion spray voltage, 5500 V for positive ion and −4500 V for negative ion ESI mode.

### 3.5. Statistical Analysis

Values were expressed as mean (RSD), unless otherwise stated. Statistical significant difference was preformed using a Student’s *t*-test, and *p* < 0.05 and *p* < 0.001 were considered statistically significant and highly significant, respectively.

## 4. Conclusions

The developed HPLC-ESI-MS/MS method exhibited excellent linearity, sensitivity, selectivity, and efficiency for the determination of total 30 active polyphenols in TMF. The coupling of HPLC separation with tandem mass spectrometry provides an attractive tool for multicomponent identification of traditional natural products. A silica-based monolithic column can efficiently facilitate a rapid separation of the complex ingredients of polyphenols. This is the first example of a comprehensive quantitation of polyphenols in TMF, which traces their origination from herbal components. Moreover, the contents of 12 flavones (**2**, **3**, **6**, **8**, **10**, **12**, **14**, **21**–**24**, and **27**) are reported for the first time in Gegen. With its high sensitivity and accuracy, the HPLC-ESI-MS/MS method can not only facilitate the quality control studies but also the further pharmacokinetic investigations of TMF [26,27] and its individual herbal components.

## Figures and Tables

**Table 1:** Thirty analyte MS/MS transitions and instrumental conditions.

A ^a^	Q_1_^b^	Q_3_^c^	DP (V)	CE (V)	A ^a^	Q_1_^b^	Q_3_^c^	DP (V)	CE (V)
1	197.0	178.9	−43	−16	16	255.1	199.1	102	35
2	579.4	255.2	62	38	17	547.3	295.0	−95	−40
3	609.3	285.2	55	31	18	547.3	295.0	−102	−41
4	137.0	108.0	−60	−32	19	192.9	133.9	−50	−22
5	417.1	255.1	55	23	20	192.9	133.9	−45	−23
6	447.3	285.2	59	22	21	447.2	285.2	58	23
7	433.3	271.2	49	23	22	563.3	311.1	−96	−42
8	565.2	271.2	70	34	23	561.3	309.1	−103	−40
9	431.1	311.0	−92	−34	24	561.3	309.1	−100	−41
10	563.2	311.1	−93	−41	25	359.1	161.0	−53	−23
11	415.1	266.9	−92	−48	26	537.3	493.2	−41	−13
12	563.3	269.2	72	30	27	577.3	325.1	−102	−40
13	445.1	325.1	−90	−34	28	717.4	519.2	−73	−26
14	475.3	313.2	75	15	29	493.1	295.0	−54	−24
15	431.3	269.2	58	23	30	267.0	251.9	−83	−30

^a^: Analyte; ^b^: precursor ion; ^c^: product ion.

**Table 2:** Contents of 22 common components presented in both Tongmai formula (TMF) and Puerariae Lobatae Radix (PLR) (*n* = 5).

Analyte	Contents/mg·g^−1^ (RSD%)		Analyte	Contents/mg·g^−1^ (RSD%)	
	TMF	PLR		TMF	PLR
**2**	0.97 ** (3.00)	1.17 (1.80)	**14**	4.41 (3.08)	4.31 (0.73)
**3**	0.30 ** (3.26)	0.36 (3.99)	**15**	0.83 (2.26)	0.84 (4.03)
**5**	7.18 * (2.74)	7.66 (2.70)	**16**	4.48 ** (3.64)	2.72 (1.06)
**6**	2.22 ** (3.84)	2.61 (2.99)	**17**	2.87 (4.41)	2.88 (4.54)
**7**	0.47 ** (1.56)	0.56 (1.98)	**18**	24.12 (4.11)	23.63 (2.64)
**8**	0.20 ** (2.20)	0.26 (4.53)	**21**	0.08 ** (3.92)	0.09 (2.65)
**9**	15.17 ** (1.78)	14.01 (1.13)	**22**	1.96 (2.13)	1.93 (4.54)
**10**	0.75 (3.17)	0.74 (4.23)	**23**	0.09 (1.15)	0.09 (2.01)
**11**	92.55 * (3.95)	83.87 (2.25)	**24**	0.37 * (3.67)	0.35 (2.70)
**12**	0.02 (1.83)	0.02 (3.39)	**27**	0.19 * (3.80)	0.18 (4.63)
**13**	12.26 * (2.44)	11.22 (3.51)	**30**	0.10 ** (1.77)	0.06 (2.76)

* *p* < 0.05 and ** *p* < 0.001, which Tongmai formula compared with Puerariae Lobatae Radix.

**Table 3:** Contents of 6 common components presented in both Tongmai formula (TMF) and Salviae Miltiorrhizae Radix et Rhizoma (SM) (*n* = 5).

Analyte	Contents/mg·g^−1^ (RSD%)		Analyte	Contents/mg·g^−1^ (RSD%)	
	TMF	SM		TMF	SM
**1**	15.24 (4.16)	15.93 (1.46)	**26**	1.67 * (3.59)	1.80 (2.97)
**4**	0.08 (2.36)	0.08 (3.94)	**28**	20.57 * (3.68)	22.45 (2.68)
**25**	0.61 * (2.97)	0.68 (4.58)	**29**	12.39 (2.18)	12.40 (2.08)

* *p* < 0.05, Tongmai formula compared with Salviae Miltiorrhizae Radix et Rhizoma.

**Table 4:** Contents of 2 common components presented in both Tongmai formula (TMF) and Chuanxiong Rhizoma (CR) (*n* = 5).

Analyte	Contents/mg·g^−1^ (RSD%)		Analyte	Contents/mg·g^−1^ (RSD%)	
	TMF	CR		TMF	CR
**19**	1.07** (1.47)	0.98 (2.99)	**20**	0.13** (1.60)	0.16 (4.26)

** *p* < 0.001, Tongmai formula compared with Chuanxiong Rhizoma.

## Supplementary Materials

Supplementary materials are available online. Table S1: Regression equation, LLOD, LLOQ, precision, repeatability and stability of the investigated compounds; Table S2: Recovery data by standard addition method (*n* = 3); Figure S1: Typical chromatograms of individual MRM transitions for Compounds **1**–**30** in the extract of Tongmai formula or Puerariae Lobatae Radix, Salviae Miltiorrhizae Radix et Rhizoma and Chuanxiong Rhizoma.
