# Supplementary material for: Rapid Determination of 30 Polyphenols in Tongmai Formula, a Combination of Puerariae Lobatae Radix, Salviae Miltiorrhizae Radix et Rhizoma, and Chuanxiong Rhizoma, via Liquid Chromatography-Tandem Mass Spectrometry
Source: Molecules. 2017 Mar 29;22(4):545. doi: 10.3390/molecules22040545 (PMC6154678; doi:10.3390/molecules22040545)
Supplement: Supplementary file 1 [file molecules-22-00545-s001.pdf]

Molecules **2017**, 22, x; doi: FOR PEER REVIEW

Supplementary Materials: Rapid Determination of 30 Polyphenols in Tongmai Formula, a Combination of Puerariae Lobatae Radix, Salviae Miltiorrhizae Radix et Rhizoma and Chuanxiong Rhizoma, by Liquid Chromatography–tandem Mass Spectrometry

Fu-Rong Wang, Ying Zhang, Xin-Bao Yang, Chun-Xu Liu, Xiu-Wei Yang, Wei Xu, Jian-Xun Liu

Table S1. Regression equation, LLOD, LLOQ, precision, repeatability and stability of the investigated compounds

A[a]	Regression equation	r	Test range (ng)	LLOD (pg)	LLOQ (pg)	Precision (RSD%)		Repeatability (RSD%)	Stability (RSD%)
						Intra-day	Inter-day		
**1**	y=5373.4x+2855.7	0.9998	2.25–45.00	19.91	66.37	0.13	1.39	1.56	1.79
**2**	y=116740x–544.03	0.9999	0.31–6.20	3.24	10.80	0.19	2.24	2.81	0.90
**3**	y =83085x–11106	0.9994	0.77–15.38	5.77	19.23	0.47	2.08	2.53	1.82
**4**	y =127833x+97570	0.9992	1.80–9.00	8.97	29.90	0.70	1.00	1.82	3.64
**5**	y=320138x+302141	0.9998	1.70–34.00	4.34	14.47	1.10	1.52	2.05	2.16
**6**	y =168195x+20572	0.9994	0.44–8.90	3.12	10.40	0.63	2.86	3.17	4.59
**7**	y=420781x+15642	0.9996	0.14–2.80	1.89	6.30	1.02	2.54	1.87	4.39
**8**	y=57266x+15400	0.9990	0.55–11.00	9.02	30.07	0.32	2.37	1.95	3.80
**9**	y=269668x+4260	0.9993	0.10–19.00	1.93	6.43	0.71	1.28	3.87	2.08
**10**	y=245082x+11500	0.9991	1.02–20.30	3.11	10.7	1.68	1.41	2.74	3.06
**11**	y=376803x+62728	0.9993	0.64–12.80	4.34	14.47	0.68	1.86	1.93	3.61
**12**	y=273630x+12782	0.9992	0.20–4.00	5.66	18.87	2.54	1.64	4.24	3.45
**13**	y=367019x+13340	0.9993	0.16–3.30	1.10	3.67	0.24	0.63	1.98	2.40
**14**	y=115479x–7207	0.9996	0.22–4.40	4.50	15.00	1.74	2.60	2.34	0.92
**15**	y=780700x+4915.4	0.9994	0.16–3.30	0.63	2.10	0.53	1.58	2.58	2.87
**16**	y=186722x+125500	0.9990	0.90–18.00	10.80	36.00	0.41	0.68	2.09	4.53
**17**	y=114349x–25254	0.9991	1.78–35.50	4.20	14.00	0.91	1.81	2.67	3.50
**18**	y=142431x+2029.4	0.9996	0.14–2.90	3.11	10.37	1.32	0.77	3.96	1.28
**19**	y=150697x+26723	0.9995	0.82–16.64	4.58	15.27	0.57	1.60	2.84	5.06
**20**	y=160472x+1375.9	0.9993	0.25–5.00	12.73	42.43	0.44	2.26	2.56	3.67
**21**	y=1000000x+6994	0.9994	0.10–4.00	1.59	5.30	0.11	1.92	2.56	2.63
**22**	y=195790x+22165	0.9995	0.55–11.00	4.12	13.73	1.90	3.09	4.34	2.06
**23**	y=155334x+3180.2	0.9990	0.12–2.50	0.51	1.70	0.62	2.09	3.46	2.64
**24**	y=221190x+17900	0.9993	0.48–9.50	2.00	6.67	2.76	3.10	3.05	0.45
**25**	y=221171x+19943	0.9998	0.83–16.64	10.80	36.00	0.33	0.89	2.81	2.50
**26**	y=41260x+1310.7	0.9991	0.20–4.00	12.63	42.10	1.19	3.16	2.79	3.55
**27**	y=277730x–1093.8	0.9991	0.06–0.82	0.78	2.60	1.80	2.60	3.25	2.42

**28**	y=63912x–860.7	0.9996	1.67–33.45	37.06	123.53	1.05	1.03	2.66	4.46
**29**	y=13038x+13194	0.9992	2.37–47.40	33.73	112.43	0.49	2.62	4.55	3.98
**30**	y=4000000x+17270	0.9996	0.04–0.90	0.69	2.30	0.42	2.07	1.72	2.80

[a] A: Analyte.

Table S2. Recovery data by standard addition method (n = 3)

Compounds	Originals (μg)	Spiked (μg)	Found (μg)	Recovery (%)	RSD (%)
		32.50	86.60	95.24	2.21
**1**	55.65	65.00	122.17	102.34	1.93
		97.50	147.20	93.90	4.38
		15.50	37.53	96.90	3.34
**2**	22.51	31.00	52.36	96.29	1.61
		46.50	66.79	95.23	2.44
		3.84	11.13	96.09	2.69
**3**	7.44	7.69	14.82	95.97	1.98
		11.53	19.12	101.30	2.79
		1.50	4.58	102.00	4.05
**4**	3.05	3.00	6.01	98.67	1.18
		4.50	7.58	100.67	1.87
		85.00	237.06	95.29	3.23
**5**	156.06	170.00	318.08	95.31	2.70
		255.00	400.02	95.67	0.60
		22.25	53.96	98.52	3.01
**6**	32.04	44.50	74.78	96.04	1.76
		66.75	97.89	98.65	3.23
		7.00	18.56	95.99	1.17
**7**	11.84	14.00	25.25	95.79	2.36
		21.00	32.97	100.62	0.85
		2.75	7.88	96.73	2.47
**8**	5.22	5.50	10.46	95.27	1.90
		8.25	13.37	98.79	1.83
		47.50	230.63	99.94	2.89
**9**	183.16	95.00	280.50	102.46	1.36
		142.50	328.93	102.29	1.16
		10.17	34.90	97.25	2.54
**10**	25.01	20.33	46.35	104.97	3.77
		30.50	56.85	104.39	3.39
**11**		320.00	1316.51	97.01	4.45
	1006.08	640.00	1677.93	104.98	0.52

		960.00	1981.08	101.56	0.91
		1.00	2.62	103.95	2.21
**12**	1.58	2.00	3.53	97.50	2.98
		3.00	4.67	103.01	1.44
		82.50	336.98	95.26	3.15
**13**	258.39	165.00	426.86	102.10	1.68
		247.50	499.49	97.41	3.45
		11.00	28.69	95.64	2.17
**14**	18.17	22.00	39.26	95.86	3.03
		33.00	51.09	99.76	1.08
		8.25	21.56	95.76	2.30
**15**	13.66	16.50	29.40	95.39	2.55
		24.75	37.90	97.94	0.97
		4.50	26.19	98.00	3.21
**16**	21.78	9.00	30.46	96.44	1.89
		13.50	35.00	97.93	1.38
		17.75	84.17	102.99	1.26
**17**	65.89	35.50	100.94	98.73	2.61
		53.25	120.15	101.91	4.85
		72.50	415.71	101.40	1.73
**18**	342.20	145.00	493.24	104.17	2.01
		217.50	565.71	102.76	1.77
		4.05	10.67	100.99	1.26
**19**	6.58	8.10	15.09	105.06	4.15
		12.15	18.66	99.42	3.57
		2.50	4.26	95.60	3.74
**20**	1.87	5.00	6.65	95.63	2.39
		7.50	9.03	95.47	4.88
		1.50	4.72	103.33	2.49
**21**	3.17	3.00	6.15	99.33	1.85
		4.50	7.78	102.44	2.34
		5.50	25.98	96.73	2.59
**22**	20.66	11.00	32.18	104.73	2.13
		16.50	37.85	104.18	1.42
		0.65	1.68	95.38	3.76
**23**	1.06	1.30	2.37	100.77	1.13
		1.95	3.09	104.10	4.38
		2.75	6.93	96.00	3.89
**24**	4.29	5.50	10.03	104.36	1.88
		8.25	12.59	100.61	0.65
**25**		2.35	6.73	97.87	1.71

	4.43	4.70	9.04	98.09	2.72
		7.05	11.53	100.71	2.24
		2.00	9.30	100.00	3.46
**26**	7.30	4.00	11.32	100.50	3.92
		6.00	13.51	103.49	4.61
		0.55	2.10	101.82	3.41
**27**	1.54	1.10	2.70	105.45	4.83
		1.65	3.19	99.98	2.62
		16.73	109.95	101.37	0.88
**28**	92.99	33.45	127.77	103.98	3.40
		50.18	142.46	98.60	2.26
		23.69	61.58	97.51	2.32
**29**	38.48	47.39	83.61	95.23	1.49
		71.08	111.03	102.07	2.67
		0.45	1.21	97.78	1.12
**30**	0.77	0.90	1.68	101.11	1.62
		1.35	2.17	103.70	2.19

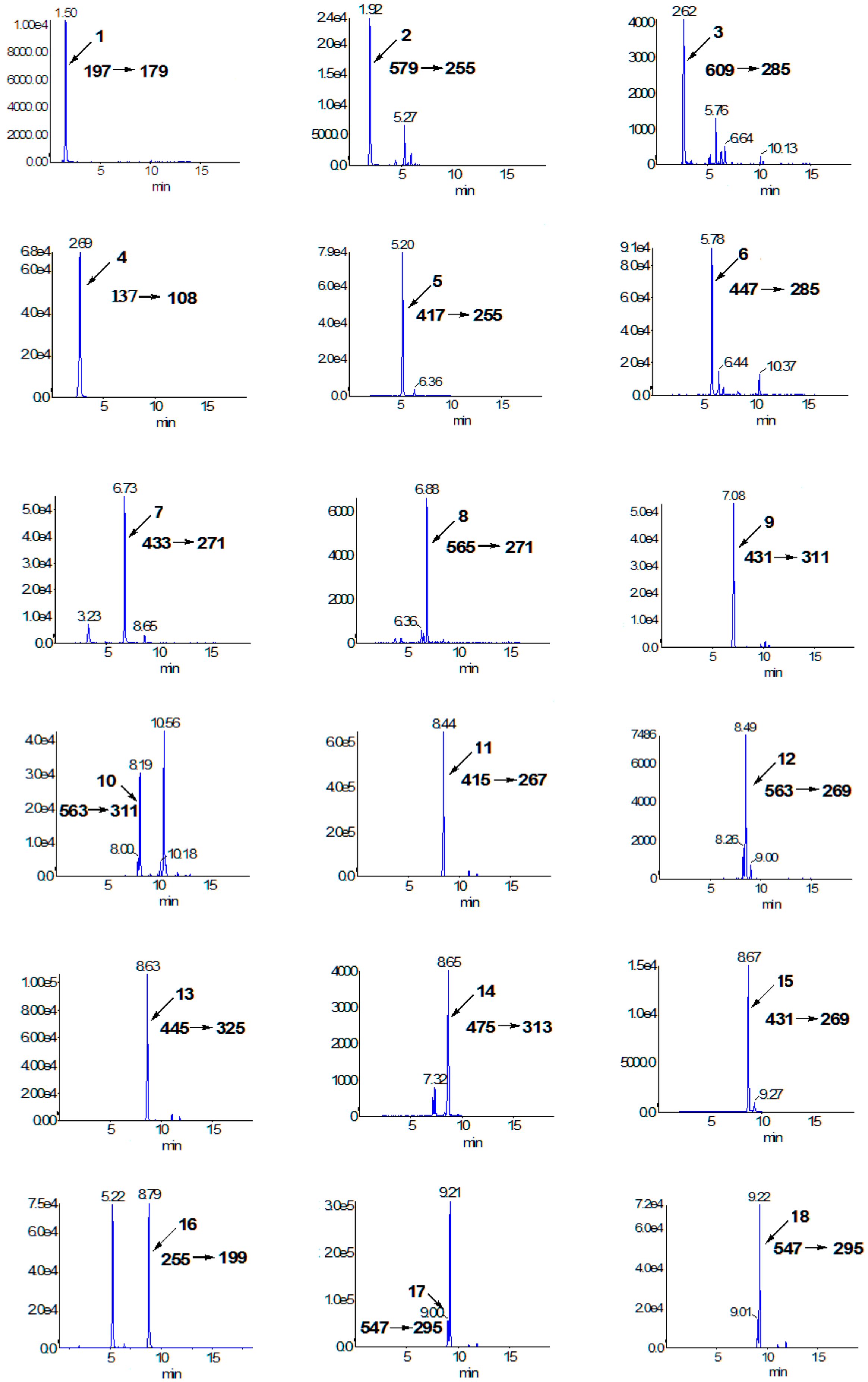
1.50
1
197 → 179
1.92
2
579 → 255
5.27
2.62
3
609 → 285
5.76
6.64
10.13
2.69
4
137 → 108
5.20
5
417 → 255
6.36
5.78
6
447 → 285
6.44
10.37
6.73
7
433 → 271
3.23
8.65
6.88
8
565 → 271
6.36
7.08
9
431 → 311
10.56
8.19
10
563 → 311
8.00
10.18
8.44
11
415 → 267
8.49
12
563 → 269
8.26
9.00
8.63
13
445 → 325
8.65
14
475 → 313
7.32
8.67
15
431 → 269
9.27
5.22
8.79
16
255 → 199
9.21
17
9.00
547 → 295
9.22
18
547 → 295
9.01
min

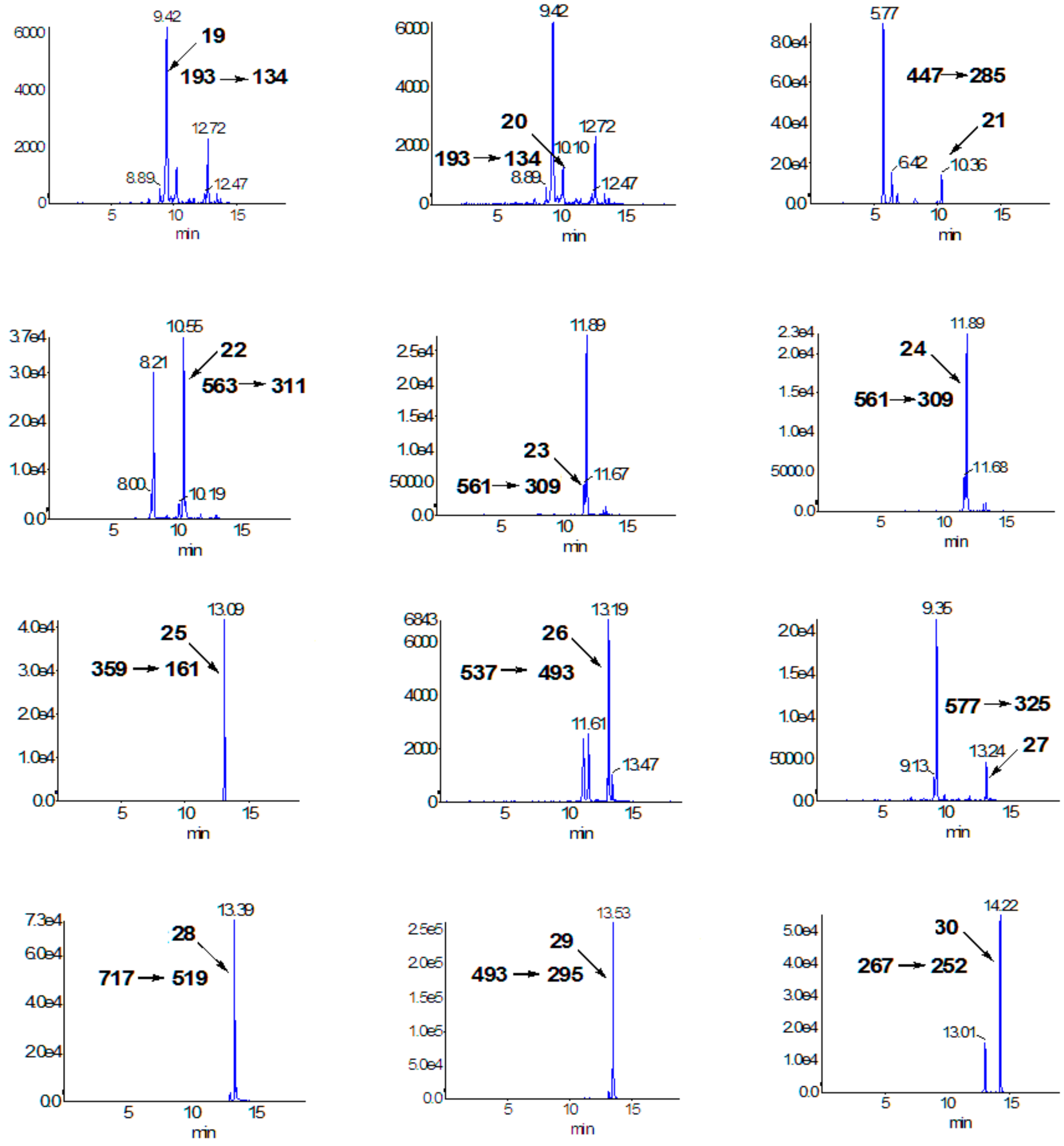

Figure S1. Typical chromatograms of individual MRM transitions for compounds **1**–**30** in the extract of Tongmai formula or Puerariae Lobatae Radix, Salviae Miltiorrhizae Radix et Rhizoma and Chuanxiong Rhizoma.
